# Comment on: Total neoadjuvant therapy *versus* standard neoadjuvant treatment strategies for the management of locally advanced rectal cancer: network meta-analysis of randomized clinical trials

**DOI:** 10.1093/bjs/znae209

**Published:** 2024-08-21

**Authors:** Corrado Pedrazzani, Giulia Turri, Giovanni Ostuzzi, Corrado Barbui

**Affiliations:** Department of Engineering for Innovation Medicine, Section of General and Hepatobiliary Surgery, University of Verona, Verona, Italy; Department of Surgical Sciences, Dentistry, Gynaecology and Paediatrics, Section of General and Hepatobiliary Surgery, University of Verona, Verona, Italy; World Health Organization Collaborating Centre for Research and Training in Mental Health and Service Evaluation, Department of Neuroscience, Biomedicine and Movement Sciences, Section of Psychiatry, University of Verona, Verona, Italy; World Health Organization Collaborating Centre for Research and Training in Mental Health and Service Evaluation, Department of Neuroscience, Biomedicine and Movement Sciences, Section of Psychiatry, University of Verona, Verona, Italy

*Dear Editor*

We congratulate Donnelly *et al.*^1^ for their effort to shed light on the debated issue of the best treatment for locally advanced rectal cancer (LARC) through a network meta-analysis (NMA) approach. In conducting a NMA, different choices might notably change the outcomes and their clinical interpretation. On this basis, we would like to point out some possible issues with this work to prompt reflection on the conclusions.

First, the included studies might not be entirely consistent with the population of interest. Specifically, the Stockholm trial recruited patients with ‘primary resectable rectal cancer’, whereas those with ‘locally advanced unresectable tumours were excluded’, and it should also be noted that early stage cancers were randomized. Indeed, baseline published characteristics only refer to the ypTNM stage, which was reported incorrectly as T stage in *Table 4*. Therefore, it may be misleading to include this trial, and we are afraid that this choice might have introduced heterogeneity and even threatened the transitivity assumption.

We also believe that the included studies do not entirely reflect the available evidence on neoadjuvant treatments for LARC. In fact, older RCTs and some new RCTs on total neoadjuvant therapy (TNT) were not included. Although it is known that some of these treatments are suboptimal, their inclusion may have been considered to increase the number of studies contributing to indirect evidence, thus increasing the precision of estimates.

Because current literature has not yet clarified the optimal TNT protocol in terms of pathological and long-term outcomes, we dispute the choice of pooling all TNT regimens together. We appreciate that the authors conducted a subgroup analysis for consolidation and induction chemotherapy, but they did not take into account the dose of radiotherapy. This might be relevant, as shown by our NMA published in JAMA Netw Open this year, as well as by the recently updated results of the RAPIDO trial, which found a significantly higher risk of locoregional recurrence with short-course radiotherapy-based TNT regimens. We believe that the effect of pooled TNT protocols might be misleading, as it precludes differentiation between the effect of different treatment regimens on survival.

Finally, the authors declared that confidence in the estimates was assessed by the CINeMA approach; however, this assessment was not reported. This severely limits our understanding of the applicability of the results to real-world practice. Specifically, statistically significant results are less credible and generalizable if obtained from indirect, heterogeneous, or biased evidence, or if pooled estimates are imprecise according to a predefined cut-off for clinical significance, which was not clearly defined in this NMA.

The authors concluded that ‘TNT regimens have a survival and recurrence benefit compared with current standards of care’ and that they ‘should represent the new standard of care in the neoadjuvant, multimodal management of LARC’. We believe that this conclusion is too categorical and should be tempered in light of the several shortcomings that we have highlighted. Although we agree with the need to incorporate TNT into clinical practice, we claim that a precise distinction between different treatment regimens, along with an accurate appraisal of their certainty, is essential to allow a safe and effective transition towards evidence-based practices.
